# Protective action of a hexane crude extract of *Pterodon emarginatus *fruits against oxidative and nitrosative stress induced by acute exercise in rats

**DOI:** 10.1186/1472-6882-5-17

**Published:** 2005-08-17

**Authors:** Fernanda BA Paula, Cibele MCP Gouvêa, Patrícia P Alfredo, Ione Salgado

**Affiliations:** 1 Departamento de Análises Clínicas e Toxicológicas, Escola de Farmácia e Odontologia de Alfenas (EFOA), Alfenas, MG, 37130-000, Brazil; 2 Departamento de Ciências Biológicas, Escola de Farmácia e Odontologia de Alfenas (EFOA), Alfenas, MG, 37130-000, Brazil; 3 Faculdade de Fisioterapia, Universidade de Alfenas, MG, 37130-000, Brazil; 4 Departamento de Bioquímica, Instituto de Biologia, Universidade Estadual de Campinas (UNICAMP), Campinas, SP, 13083-970, Brazil

## Abstract

**Background:**

The aim of the present work was to evaluate the effect of a hexane crude extract (HCE) of *Pterodon emarginatus *on the oxidative and nitrosative stress induced in skeletal muscle, liver and brain of acutely exercised rats.

**Methods:**

Adult male rats were subjected to acute exercise by standardized contractions of the tibialis anterior (TA) muscle (100 Hz, 15 min) and treated orally with the HCE (once or three times with a fixed dose of 498 mg/kg), before and after acute exercise. Serum creatine kinase activity was determined by a kinetic method and macrophage infiltration by histological analyses of TA muscle. Lipid peroxidation was measured as malondialdehyde (MDA) levels. Nitric oxide production was evaluated by measuring nitrite formation, using Griess reagent, and nitrotyrosine was assessed by western blotting.

**Results:**

Serum creatine kinase activities in the controls (111 U/L) increased 1 h after acute exercise (443 U/L). Acute exercise also increased the infiltration of macrophages into TA muscle; lipid peroxidation levels in TA muscle (967%), liver (55.5%) and brain (108.9%), as well as the nitrite levels by 90.5%, 30.7% and 60%, respectively. The pattern of nitrotyrosine formation was also affected by acute exercise. Treatment with HCE decreased macrophage infiltration, lipid peroxidation, nitrite production and nitrotyrosine levels to control values.

**Conclusion:**

Acute exercise induced by functional electrical stimulation in rats resulted in increase in lipid peroxidation, nitrite and nitrotyrosine levels in brain, liver and skeletal muscle. The exercise protocol, that involved eccentric muscle contraction, also caused some muscle trauma, associated with over-exertion, leading to inflammation. The extract of *P. emarginatus *abolished most of these oxidative processes, thus confirming the high antioxidant activity of this oil which infusions are used in folk medicine against inflammatory processes.

## Background

Although exercise has salutary effects, strenuous or unaccustomed physical exercise can cause morphological insult to the myocardial and skeletal muscle involved in the activity, as well as to others organs [1]. Tissue damage resulting from acute or chronic exercise ranges from considerable fiber disruption to subcellular damage [2,3]. Such damage may arise from oxidative stress caused by reactive oxygen species (ROS), which elicit different responses, depending on the organ or tissue and its levels of endogenous antioxidant [1]. In response to eccentric contraction-induced muscle damage, neutrophils and macrophages migrate to the site, infiltrate the muscle tissue, activate cytokines, and produce additional ROS [4,5]. These activities may overwhelm the natural antioxidant defenses of the cell and lead to lipid peroxidation and delayed-onset muscle damage [4,5]. Increased levels of lipid peroxidation have been detected after sprint exercise in blood samples from sprint-trained athletes [6] and in the skeletal muscle of rats after sprint and acute exercise [3,5].

Acute exercise also increases nitric oxide (NO) formation and NO synthase (NOS) activity [7,8]. Reid [9] proposed that, in addition to ROS, NO may play an important role in skeletal muscle, and that muscles involved in repetitive contraction may produce sufficient NO to influence the oxidant-antioxidant balance. NO has been implicated in the metabolic control of muscle by its effects on blood delivery, glucose uptake, oxidative phosphorylation, contractility, and excitation-contraction coupling [10]. However, exposure to reactive oxygen and nitrogen species (RONS) may cause lipid peroxidation in cell membranes, which in turn may generate species that damage cell proteins and promote their degradation [11]. These actions may impair the performance, integrity and metabolism of muscle cells [4]. In addition to the increase in lipid peroxidation following acute exercise, the effects of nitration need to be considered, especially since tyrosine nitration is frequently linked to altered protein function during inflammatory conditions [12]. Protein nitration has been suggested to be a final product of the highly reactive nitrogen oxide intermediates(*e.g. *peroxynitrite) formed in reactions between NO and oxygen-derived species such as superoxide. However, Gunther et al. [13] described a mechanism for NO-dependent tyrosine nitration that does not require the formation of highly reactive nitrogen oxide intermediates such as peroxynitrite or nitrogen dioxide. Peroxidases can also oxidize nitrite to nitrogen dioxide radical, which can cause nitration of tyrosine and tyrosine residues in proteins [14].

*Pterodon emarginatus *Vog. (Leguminosae, Papilonaceae), popularly known as sucupira branca, is a native tree species widely distributed throughout central Brazil, in the states of Goiás, Minas Gerais and São Paulo. Sucupira infusions are used in folk medicine for the treatment of rheumatism, sore throats and back-bone problems [15]. *Pterodon *seed oil has cercaricidal activities [16], an acute anti-inflammatory action in rats [17] and an analgesic effect in mice [18]. The seed oil contains diterpenes and furanditerpenes [19]. The most ubiquitous furanditerpene of *P. emarginatus *fruits is 6α,7β-di-hydroxyvouacapan-17β-oic acid [19], the anti-inflammatory activity of which has been evaluated in rodent experimental models of paw edema induced by carrageenin and nystatin [17,20].

Although there have been a number of reports on the anti-inflammatory activity of *Pterodon *oil, there has been no investigation of its antioxidant activity. The aim of the present work was to investigate the antioxidant activity and the effect on nitrite production of hexane crude extract of *P. emarginatus *fruits in acutely exercised rats.

## Methods

### Animals

The present study is in compliance with the principals and guidelines of the Brazilian Institute for Animal Experimentation (COBEA) and it had institutional ethical approval by the Ethics Committee for Animal Research, Escola de Farmácia e Odontologia de Alfenas/Centro Universitário Federal (Efoa/Ceufe; Permission: 15/04/2004).

Male Wistar rats (270 ± 20 g) were obtained from the Efoa/Ceufe. The rats were housed in a temperature-controlled room on a 12 h light/dark schedule with food and water available *ad libitum*. The rats were allocated to six experimental groups: (1) rested control group; (2) acutely exercised group; (3) rested group treated with *P. emarginatus *hexane crude extract (HCE); (4) acutely exercised group treated with the *P. emarginatus *HCE 0.5 h before exercise, (5) acutely exercised group treated with the *P. emarginatus *HCE 0.5 h after exercise and (6) acutely exercised group treated with three administrations of HCE given at 24 h, 12 h and 0.5 h before exercise. The rats were sacrificed 1 h, 6 h and 48 h after HCE administration (group 3) or after acute exercise (groups 2, 4–6). For the determination of CK levels the rats were sacrificed immediately or 1 h, 6 h and 48 h after exercise. For each treatment, 8–10 rats were used, as specified in the figure legends.

### Acute exercise

The rats were acutely exercised using functional electrical stimulation to produce standardized repetitive activation of the fast tibialis anterior (TA) muscle, according to Matsuura et al. [21] with modifications. Briefly, the animals were boxed and two square electrodes (1 cm^2^) were then fixed on the skin of their right legs at proximal and distal ends of the TA muscle. TA muscle was stimulated electrically (Neurodyn II-Ibramed) through an electrode with 10 s square wave biphasic pulses and interpulse intervals of 10 s of 5 V for 10 min. Stimulation frequency was 100 Hz, with an on-off ratio of 1:1.

### Plant extract preparation and administration to rats

*Pterodon emarginatus *Vog. fruits were harvested in the state of Minas Gerais, Brazil. A crude hexane extract (HCE) was prepared according to Carvalho et al. [20]. Briefly, the fruits were ground in the presence of hexane using an industrial blender and then placed in a percolator at room temperature and successively washed with hexane. The extraction liquid was collected, filtered and concentrated by rotatory evaporation under reduced pressure. The resulting HCE had an oily characteristic and a density of 0.98 g/mL and was administered orally (by gavage) to the rats, in one or three doses at 498 mg/kg each.

### Serum creatine kinase (CK) activity and lactate level determinations

Blood samples were obtained by cardiac punction from anesthetized rats either before or after the acute exercise. The serum CK activity was determined by a colorimetric assay (CK-NAC kit, Bayer), at 340 nm, and was expressed in U/L, where 1 U resulted in the phoshorylation of 1 mmol of creatine per min at 25°C. Serum lactate concentrations were determined by a colorimetric assay at 550 nm, using lactate oxidase [22].

### Histological analysis

The tibialis anterior (TA) muscle was removed from all rats and fixed in 4% (v/v) formalin, embedded in paraffin, cut longitudinally into 5 μm sections and stained with hematoxylin-eosin. The density of macrophages and the extent muscle fiber damage were estimated by point counting morphometry, using 15 randomly selected fields of 0.625 mm^2 ^per section.

### Measurement of TBARS in brain, liver and TA muscle

As an index of oxidative damage, lipid peroxidation was evaluated by using the level of thiobarbituric acid-reactive substances (TBARS) test [23]. Brain, liver and TA muscle were homogenized in four volumes of 0.1 M phosphate buffered saline (PBS) and centrifuged at 3,000 *g*, 4°C, for 10 min. Aliquots (0.5 ml) of each homogenate were mixed with 0.5 mL of a solution containing 2% thiobarbituric acid, 0.5 mL of 25% HCl and 45 μL of 2% BHT (butylated hydroxytoluene). The mixtures were heated at 95°C for 10 min and then centrifuged. The supernatant was transferred to a quartz cuvette and the absorption was measured at 532 nm. The absorption values were transformed into concentrations of MDA using 1,1,3,3-tetraethoxypropane (TEP) as standard.

### Determination of nitrite in brain, liver and TA muscle

Nitrite is a stable end product of NO, and its concentration was determined by the Griess method [see [24]]. Brain, liver and TA muscle were homogenized in four volumes of 30 mM Tris-HCl buffer, pH 6.8, containing 5 mM EDTA, 250 mM sucrose, 30 mM KCl, 2% β-mercaptoethanol, PMSF (100 μg/mL), benzamidine (5 μg/ml), aprotinin (2 μg/mL) and leupeptin (2 μg/mL), and then centrifuged (12,000 × *g*, 4°C, 15 min). Protein concentrations were determined by the Coomassie blue dye-binding method [25] using bovine serum albumin as standard. Aliquots (1.0 mL) of the homogenates were removed and diluted with 1.0 mL of Griess reagent (1% sulphanilamide, 2% phosphoric acid and 0.1% naphthyl ethylene diamine dihydrochloride). The absorbance of the chromophore formed during diazotization of the nitrite with sulphanilamide and subsequent coupling with naphthylethelene diamine was measured at 545 nm. Appropriate blanks and controls were run in parallel.

### Western blotting analysis

Aliquots (30 μg) of protein from brain, liver and TA muscle were run on 9% gels using sodium dodecyl sulfate-polyacrylamide gel electrophoresis and were electrophoretically transferred overnight, at 4°C, to nitrocellulose membranes in 20 mM Tris, 192 mM glycine and 20% methanol. The membranes were blocked for 1 h at 4°C in Tris-buffered saline containing 30% Tween 20 and 50% Triton X-100 (TBSTT) with 1% BSA. The blots were then incubated for 2 h with anti-nitrotyrosine antibody (1:1000) (Sigma) in TBSTT with 1% BSA at room temperature. The membranes were washed five times in TBSTT with 0.1% BSA, and incubated for 1 h with peroxidase-conjugated anti-goat IgG (1:2500) (Amersham) in TBSTT with 1% BSA at room temperature. The blots were again washed five times in TBSTT with 0.1% BSA and then visualized using an Enhanced Chemiluminescence (ECL) detection system (Amersham) [26].

### Statistical analysis

The results were expressed as the means ± SE of the number of measurements (independent experiments) shown. Statistical comparisons were done using ANOVA and the Tukey-Kramer test, with *P*<0.05 indicating significance.

## Results

### Acute exercise increases serum creatine kinase activity

**Figure 1:**
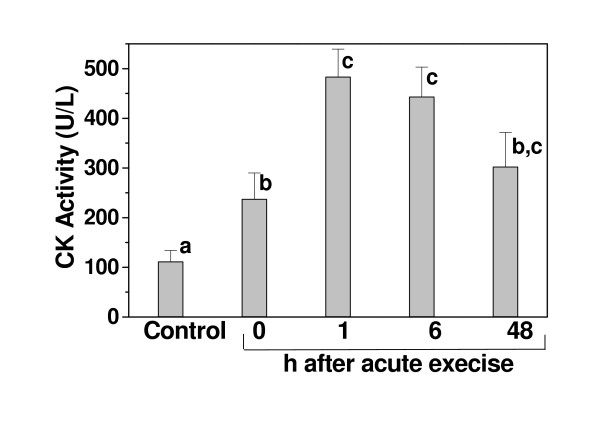
**Effect of acute exercise on serum creatine kinase (CK) activity**. The CK levels were determined in rested control and after acute exercise, at the indicated times. The results are the mean ± SE of 8–10 rats per treatment. Significance was determined with one-way ANOVA followed by the Tukey-Kramer test. Columns with different letters differed significantly (p < 0.05).

Serum creatine kinase (CK) activity increased in exercised rats. Maximum CK activity was seen 1 h after acute exercise and remained elevated for up 48 h after exercise (Fig. 1). The serum lactate concentration also increased in the exercised rats (75.36 ± 1.48 mg/dL, n = 8) when compared to the controls (54.67 ± 3.06 mg/dL, n = 8).

### HCE reduces macrophage infiltration in TA muscle induced by acute exercise

**Figure 2:**
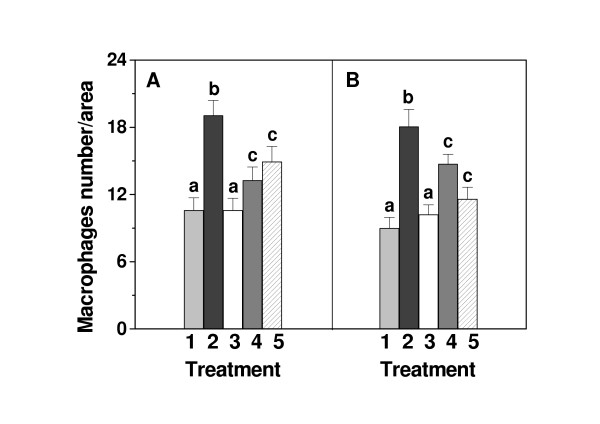
**Effect of HCE (498 mg/Kg), administrated orally to acute exercised rats, on the density of macrophages in the tibialis anterior muscle**. The number of cells per 0.625 mm^2 ^area was determined in muscles of rested control rats (1); acutely exercised rats (2); rested rats treated with HCE (3); acutely exercised rats treated with HCE 0.5 h before exercise (4) and acutely exercised rats treated with HCE 0.5 h after exercise (5). The number of cells was determined at 6 h (A) and 48 h (B) after acute exercise. Each column shows the mean ± SE of 15 randomly selected fields of 8–10 rats. Significance was determined with one-way ANOVA followed by the Tukey-Kramer test. Columns with different letters differed significantly (p < 0.05).

As shown in Fig. 2, intense exercise increased the infiltration of macrophages into the tibialis anterior (TA) muscle. Higher density was already observed 6 h after exercise and after 48 h it was still elevated. For our study, we have chosen an exercise protocol that involved a polymetric (eccentric) muscle component. Untrained exercised rats therefore experience both oxidative and polymetric stress, with the latter causing some muscle trauma, associated with over-exertion, leading to inflammation. Oral administration of the HCE alone did not affect the density of macrophages in TA muscle. However, the increased density of macrophages, seen in TA muscle of acutely exercised rats was significantly reduced, when the HCE was administered 0.5 h before or 0.5 h after acute exercise. When the HCE was administered 0.5 h after acute exercise, cell density of macrophages reduced to almost control values 48 h after exercise indicating its effectiveness against the delayed-onset of muscle trauma.

### HCE reduces lipid peroxidation in TA muscle, brain and liver induced by acute exercise

**Figure 3:**
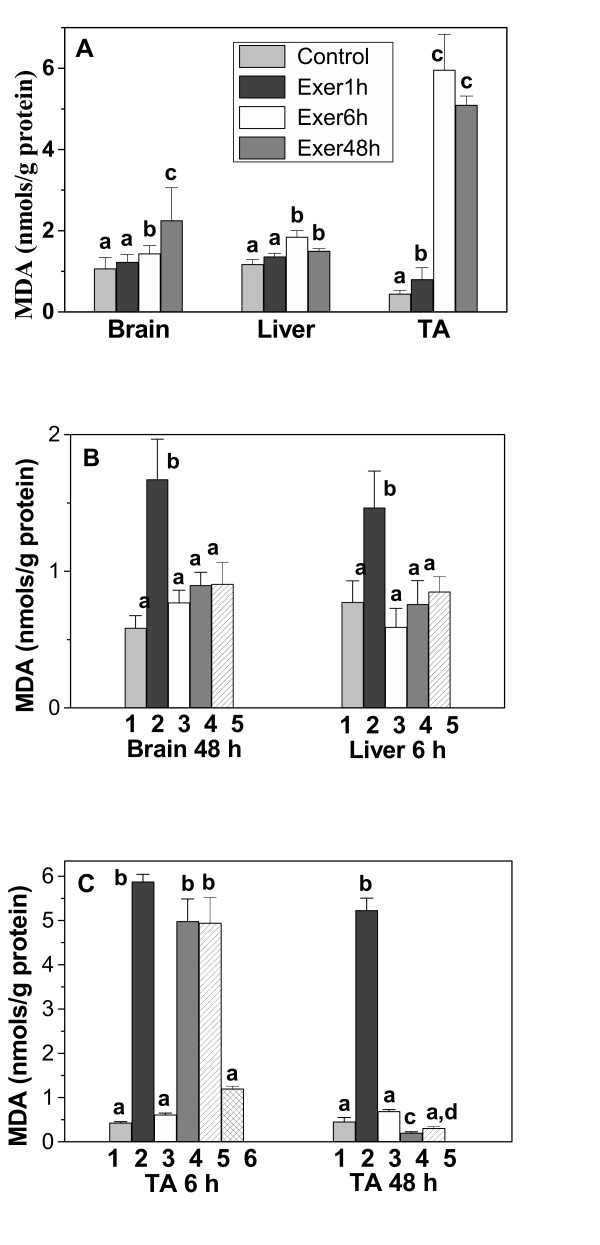
**Effect of the HCE from *P. emarginatus *on brain, liver and TA muscle lipid peroxidation after acute exercise**. (A) TBARS were determined in tissue homogenates of rested control rats and 1 h (Exer 1 h), 6 h (Exer 6 h) and 48 h (Exer 48 h) after acute exercise. TBARS were determined in brain and liver (B) and in TA muscle (C) homogenates of rested control rats (1); acutely exercised rats (2); rested rats treated with HCE (3); acutely exercised rats treated with HCE 0.5 h before exercise (4), acutely exercised rats treated with HCE 0.5 h after exercise (5) and acutely exercised rats treated with HCE 24, 12 and 0.5 h before exercise (6). TBARS levels were estimated at 48 h (brain), 6 h (liver) and 6 h and 48 h (TA muscle) after exercise, as indicated. Each column shows the mean ± SE of 8–10 rats. Significance was determined with one-way ANOVA followed by the Tukey-Kramer test. Columns with different letters differed significantly (p < 0.05).

Lipid peroxidation, evaluated by TBARS formation (MDA), was assessed in brain, liver and tibialis anterior muscle. Lipid peroxidation increased after acute exercise in all of the tissues analyzed (Fig. 3A). The highest TBARS formation was seen in TA muscle, with an increase of 967%, followed by brain (109%) and liver (55.5%). The time at which maximum lipid peroxidation was seen after exercise differed among the tissues – 48 h in brain and 6 h in liver and TA muscle. In TA muscle, the MDA levels were still elevated after 48 h (Fig. 3A).

Based on the results shown in figure 3A, we studied the effects of the HCE on lipid peroxidation in TA muscle 6 h and 48 h after acute exercise. In brain and liver, the effects of HCE were analyzed at 48 h and 6 h after acute exercise, respectively. The effect of HCE on lipid peroxidation was assessed by administering the extract 0.5 h before and 0.5 h after acute exercise. These treatments completely decreased lipid peroxidation in brain and liver tissues (Fig. 3B). However, in TA muscle, the HCE was effective in reducing lipid peroxidation only 48 h after acute exercise; the levels of MDA were still elevated 6 h after acute exercise (Fig. 3C). Then we examined the effect of three administrations of a fixed dose (498 mg/Kg) of HCE given 24 h, 12 h and 0.5 h before acute exercise. This protocol significantly reduced the TBARS in TA muscle 6 h after exercise (Fig. 3C), indicating that this treatment was more effective in reducing the lipid peroxidation induced by acute exercise in TA muscle, when compared with a single treatment protocol.

### HCE reduces nitric oxide levels in TA muscle, brain and liver increased by acute exercise

**Figure 4:**
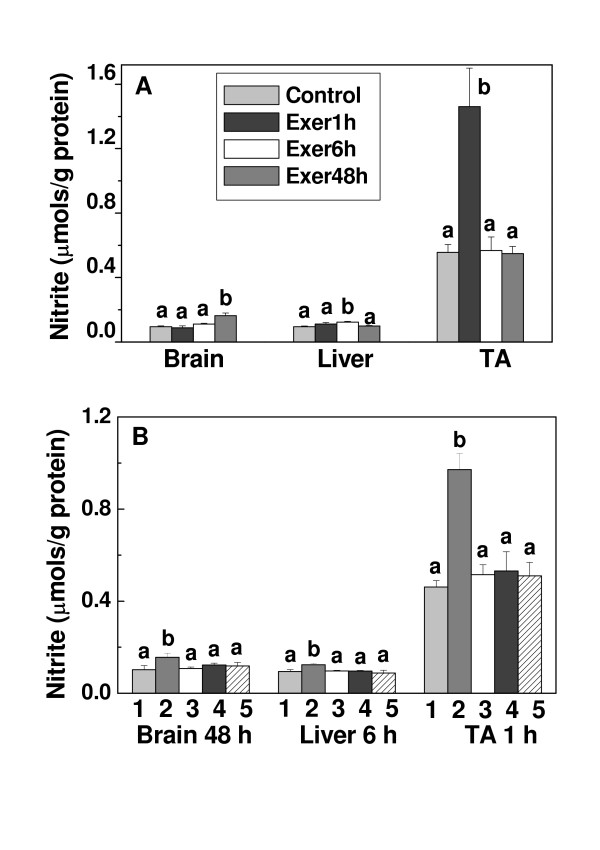
**Effect of the HCE from *P. emarginatus *on nitrite levels in brain, liver and TA muscle after acute exercise**. (A) Nitrite levels were estimated by the Griess reaction in tissue homogenates of rested control rats and at 1 h (Exer 1 h), 6 h (Exer 6 h) and 48 h (Exer 48 h) after exercise. (B) Nitrite was determined in tissue homogenates of rested control rats (1); acutely exercised rats (2); rested rats treated with HCE (3); acutely exercised rats treated with HCE 0.5 h before exercise (4) acutely exercised rats treated with HCE 0.5 h after exercise (5). Nitrite levels were estimated at 1 h (TA muscle), 6 h (liver) and 48 h (brain) after exercise. Each column shows the mean ± SE of 8–10 rats. Significance was determined with one-way ANOVA followed by the Tukey-Kramer test. Columns with different letters differed significantly (p < 0.05).

As shown in Fig. 4A, the basal nitrite concentration in TA muscle was seven and six times greater than in brain and liver, respectively. Despite this difference, acute exercise increased the nitrite production by 60.0%, 30.7% and 90.5% in brain, liver and TA muscle, respectively. The maximum nitrite levels in brain, liver and TA muscle were observed 48 h, 6 h and 1 h after exercise, respectively (Fig. 4A). When HCE was administered 0.5 h before or 0.5 h after acute exercise, the nitrite production decreased to basal levels in all of the tissues (Fig. 4B). The effects of the HCE on nitrite production were assessed at 1 h (TA muscle), 6 h (liver) and 48 h (brain) after exercise because at those times we found the maximal nitrite production in the respective tissues (Fig. 4A). The effect of HCE was similar, regardless of whether it was administered three times or only once (data not shown).

### HCE prevents nitration of brain and muscle proteins increased by acute exercise

**Figure 5:**
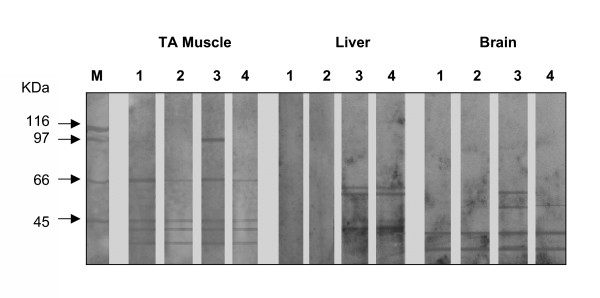
**Western blot analysis for nitrotyrosine formation in brain, liver and TA muscle**. M, molecular mass markers. Lane 1, rested control rats. Lane 2, rested control rats treated with HCE. Lane 3, acutely exercised rats. Lane 4, acutely exercised rats treated with HCE 24, 12 and 0.5 h before acute exercise. Nitrotyrosine formation was analyzed at 1 h (TA muscle), 6 h (liver) and 48 h (brain) after exercise. The results are representative of three independent experiments.

Western blot analysis using an antibody specific for nitrotyrosine revealed different staining patterns for brain (48 h after exercise), liver (6 h after exercise) and TA muscle (1 h after exercise) that were consistent with the nitration of one or more tyrosine residues on proteins (Fig. 5). In the brain, two more bands (66 and 51 kDa) were detected in the exercised rats. The administration of three doses of HCE (498 mg/kg each), given 24 h, 12 h and 0.5 h before acute exercise, prevented the formation of these bands, indicating that the extract prevented exercise-induced nitrotyrosine formation in the brain. In liver, no nitrotyrosine was detected in the control or HCE administered rats, but in exercised rats three bands (66, 55 and 45 kDa) were evident and the treatment with HCE did not prevent nitrotyrosine formation in this tissue. In TA muscle, a triplet band pattern and a band of 66 kDa were seen in the control. These bands were less intense in the HCE administered rats. The exercised rats showed an intense band at 97 kDa, in addition to the 66 kDa band and the triplet. In TA muscle from HCE-exercised rats, labeling of the 97 kDa was abolished and the intensity of the triplet decreased, indicating that the extract prevented nitrotyrosine formation. The 97 kDa band can be related to phagocyte activity in the TA muscle.

## Discussion

In this work we examined the effects of an HCE from *Pterodon emarginatus *on the oxidative and nitrosative stress induced by acute exercise. Whereas a large number of studies have tested the effects of vitamins and minerals in preventing oxidation in exercise, there is little information on the effect of plant extracts on lipid peroxidation and nitric oxide formation during acute exercise.

An increase in serum CK and in neutrophil and macrophage infiltration after acute exercise has been reported in humans and animals [27-29]. However, there is controversy about the relationships between CK kinetics and the time at which the most prominent muscle cell damage occurs. The degree of muscle damage is considered to be proportional to muscle loading [4]. Our results showed that CK activity increased after acute exercise, with maximum activity after 1 h and remained elevated for at least 48 h. The number of inflammatory cells increased after 6 h and also persisted for more than 48 h. The HCE prevented the muscle fiber damage induced by electrical stimulation, regardless of whether it was given before or after the acute exercise, and this was reflected in the decrease in macrophage density in the HCE administered rats.

Acute exercise induced the lipid peroxidation in all of the tissues studied, although there were differences in the kinetics of lipid peroxidation among the tissues. These differences have previously been described and it appears to be dependent on factors such as oxygen consumption, oxidant susceptibility and the activation of antioxidant enzymes and other repair systems [1]. Increased levels of lipid peroxidation have also been detected in the skeletal muscle of rats after acute [3] and long-term submaximal [4] exercise. However, supramaximal exercise may produce more ROS and be more damaging. In fact there is a larger increase in TBARS after high-intensity exercise in which lactate production is substantial when compared with moderate-intensity exercise [3,30]. Moreover, a significant relationship between the plasma lactate concentration and lipid peroxidation during progressive incremental exercise has been reported [31]. In agreement with this, lactate increases hydroxyl radical generation by the Fenton reaction resulting in lipid peroxidation [32]. Thus, the increase in lactate concentration seen here after acute exercise could account for the increase in TBARS levels detected in skeletal muscle.

The TBARS also increased in liver after 6 h and in brain after 48 h of acute exercise. In contrast, Kayatekin et al. [33] found that TBARS levels in the liver were unchanged after acute sprint exercise. Part of the reason for these contradictory findings could be attributed to the use of different types and intensities of exercise.

Our results also showed that lipid peroxidation induced by acute exercise were markedly higher in brain than in liver. In fact brain tissue has high content of oxidizable substrates, such as polyunsaturated fatty acids, poor catalase activity and low iron-binding capacity making it particularly prone to oxidative stress damage [1].

Although the HCE from *P. emarginatus *reduced TBARS formation in all of the tissues, it was most effective in TA muscle when administered at three different times before exercise. Thus, the decrease in MDA levels seen after administration of the HCE indicates that the extract has antioxidant activity. The higher level of HCE needed to prevent lipid peroxidation in TA muscle agreed with the greater levels of lipid peroxidation seen in this organ after electrical stimulation.

Nitrite is a stable metabolite of NO and can be used as an indicator of the overall formation of NO *in vivo*. Increased nitrite levels as a result of increased NOS activity have been observed in skeletal muscle after contractile activity [34]. In our study, acute exercise increased the nitrite levels in all of the tissues. In TA muscle, an increase on nitrite production and lipid peroxidation occurred within 1 h after acute exercise. However, in brain and liver, the increase in the levels of nitrite occurred at 48 h and 6 h after acute exercise, respectively and the lipid peroxidation increased after 6 h in the brain and liver. These findings indicate that despite the differences in time response of the organs to acute exercise, all of them suffered oxidative and nitrosative stress. Additionally, trauma caused by acute exercise in muscle was shown to be transferable to other organs. However, the highest levels of these compounds were detected in TA muscle, probably because it was the direct site of injury.

Our results also show that the basal nitrite level in TA muscle was five times greater than in brain and liver. This could be partly explained by the fact that skeletal muscle has a relatively high NOS activity compared with other organs [35]. Acute exercise increases NOS activity [8] and thus NO formation [7] in muscle. The increased nitrite formation seen after exercise could be mediated by increased intracellular calcium content of muscle fibers during contraction, which would activate NOS. Consistent with this is the observation that both of the constitutively expressed isoforms of NOS (eNOS and nNOS) require calcium as a cofactor for activation [36], and there is evidence that extracellular calcium may also enhance NOS activation during increased loading [37]. Thus, as calcium enters the sarcoplasm, not only does it induce muscle contraction, but it also activates calcium-dependent, constitutively expressed isoforms of NOS.

The HCE reduced nitrite production in TA muscle when administered before or after acute exercise. The extract was also effective in liver and brain tissues, where it decreased the nitrite formation induced by acute exercise. Our results for lipid peroxidation and nitrite formation agree with previous reports, indicating that the rate of NO production in rat liver is similar to the rate of superoxide anion production [38].

Determination of the protein nitrotyrosine content is frequently used to detect oxidative damage to tissues. Protein nitration has been suggested to be a final target of highly reactive nitrogen oxide intermediates (e.g. peroxynitrite) formed in reactions between NO and oxygen-derived species such as superoxide. Since NO is made by a variety of cell types, the opportunity for the formation of a tyrosine iminoxyl free radical exists in any protein in which tyrosyl radical formation occurs [13]. Additionally, myeloperoxidase and peroxidase can also oxidize nitrite to nitrogen dioxide radicals, which can participate in the nitration of tyrosine residues in protein [14]. Western blotting showed more than one band of nitrotyrosine in proteins in all of the tissues analyzed. Acute exercise increased nitrotyrosine formation in the tissues and HCE prevented the nitration of brain and skeletal muscle proteins. The reduction in nitrite levels and nitrotyrosine formation by HCE indicated that the extract prevented the production of reactive nitrogen species generated from the activity of NOS after acute physical exercise. In liver the HCE had no effect on protein nitration suggesting that, at least, part of the RNS formation and protein nitration in muscle are linked to phagocyte activity. As the HCE presented anti-inflammatory activity, it seemed also to prevent the secondary oxidative damage, caused by activated phagocytes.

The antioxidant action of the HCE could be partly explained by its terpene and phenol content [19]. Studies of the inhibition of lipid peroxidation have demonstrated a role of terpens and phenols [39,40]. Phenolic compounds have a high reactivity towards lipid peroxyl radicals and are thus able to interact with ROS to interrupt the propagation of lipid peroxidation [40]. Di Mascio et al. [41] showed that furan diterpenes isolated from an alcoholic extract of Pterodon sp had a high capacity to quench oxygen singlets but not to scavenge oxyradicals since the extract did not inhibit microssomal lipid peroxidation. A diterpene isolated of the plant *Salvia miltiorrhiza *was shown to inhibit lipid peroxidation in rat kidney and brain homogenates, and its activity was attributed to the presence of the furan ring in its structure [39]. In mouse brain, plant extracts containing polyphenols inhibited NMDA-induced lipid peroxidation and restored the glutathione levels [42].

Recently, nNOS was found to accommodate phenolic substituted compounds in its active site, indicating that such substances could act as inhibitors of nNOS and also as antioxidants [43]. Since the HCE contains phenolic constituents, this could explain the ability of the extract to inhibit nitrite formation in brain and muscle homogenates.

## Conclusion

In conclusion, acute exercise induced by functional electrical stimulation in rats resulted in increase in TBARS, nitrite and nitrotyrosine levels in brain, liver and skeletal muscle and macrophage infiltration in muscle fibers. The HCE of *P. emarginatus *abolished most of these oxidative processes, thus providing for a high antioxidant and anti-inflammatory action against acute exercise. Despite the well-documented protective role of plant antioxidant substances and their ability to protect against the deleterious effects of oxidation, most studies have examined the antioxidant effect of only one class of compound or of an isolated compound, mainly *in vitro*. Our results showed that a crude extract of *P. emarginatus *had anti-inflammatory, antioxidant and anti-nitrosative activities *in vivo *in various organs following acute exercise. Crude plant homogenates are generally less expensive to obtain and more accessible to the population than an isolated compound. Thus, our results support the beneficial effects of the *Pterodon *extract used popularly, and suggest its potential use in humans as a therapeutic agent against oxidative damage.

## Competing interests

The author(s) declare that they have no competing interests.

## Authors' contributions

FBAP carried out all of the experiments and participated in the drafting of the manuscript, PPA participated in the experiments of histological analysis, CMCPG supervised the work of FBAP and PPA, participated in the design drifting of the manuscript and IS coordinated the study and drafted the manuscript. All authors read and approved the final manuscript.

## Pre-publication history

The pre-publication history for this paper can be accessed here:


